# Insights into pyrazinone biosynthesis from biochemical and cryo-EM studies of the hybrid NRPS-PKS enzyme CorA

**DOI:** 10.1016/j.engmic.2026.100308

**Published:** 2026-10-08

**Authors:** Qingyu Yang, Qian Wang, Xiaoju Li, Moli Sang, Tiandong Hu, Degao Wang, Lele Zhu, Dalei Wu, Yuezhong Li, Shengying Li, Changsheng Wu, Hang Zhang, Wei Zhang

**Affiliations:** a State Key Laboratory of Microbial Technology, Shandong University, Qingdao, Shandong, 266237, China; b Laboratory of Experimental Marine Biology, Shandong Province Key Laboratory of Marine Biodiversity and Bio-resource Sustainable Utilization, Institute of Oceanology, Chinese Academy of Sciences, Qingdao, 266000, China; c Laboratory for Marine Biology and Biotechnology, Qingdao Marine Science and Technology Center, Qingdao, Shandong, 266237, China; d University of Chinese Academy of Sciences, Beijing, 100049, China; e Shandong Provincial Key Laboratory of Deep Sea Bioresources Exploration and Utilization, Shandong University, Qingdao, 266237, China

**Keywords:** NRPS-PKS hybrid, Acyltransferase, Ketosynthase, Cryo-EM structure, Pyrazinone biosynthesis

## Abstract

Hybrid nonribosomal peptide synthetase-polyketide synthase (NRPS-PKS) systems integrate peptide and polyketide biosynthetic logic to assemble complex natural products. CorA, a prototypical NRPS-PKS hybrid from the myxobacterium *Corallococcus exiguus* SDU70, is responsible for producing 5-alkylated pyrazinones, a family of bioactive metabolites with morphogenetic roles in myxobacterial development. Previous studies have identified CorA as a core biosynthetic enzyme responsible for the formation of 5-methylated pyrazinones; however, the structural and mechanistic basis underlying its capacity to recognize diverse dipeptide substrates and accommodate distinct acyl-CoA extender units remains elusive. Here, combining *in vitro* biochemistry, structure determination by cryo-electron microscopy (EM), mutagenesis, and molecular docking, we show that full-length CorA generates twelve distinct 5-alkylated pyrazinone products using six amino acid combinations and two acyl-CoA extenders, highlighting an unusually permissive gating mechanism within the KS domain. The 3.1-Å cryo-EM structure of the CorA KS-AT didomain provides structural insights into its substrate-binding architecture. The atypical SGFH motif of the AT domain permits dual binding of M-CoA and MM-CoA, and an irregular non-helical loop in the KS domain creates an open pocket for large amino acid-derived intermediates. These distinctive architectural features confer robust substrate promiscuity on CorA. Collectively, our structural data offers insights to understand the wide substrate compatibility of NRPS-PKS hybrid enzymes and may offer useful clues for the rational engineering of biosynthetic pathways to diversify natural product scaffolds.

## Graphical abstract

**Figure:**
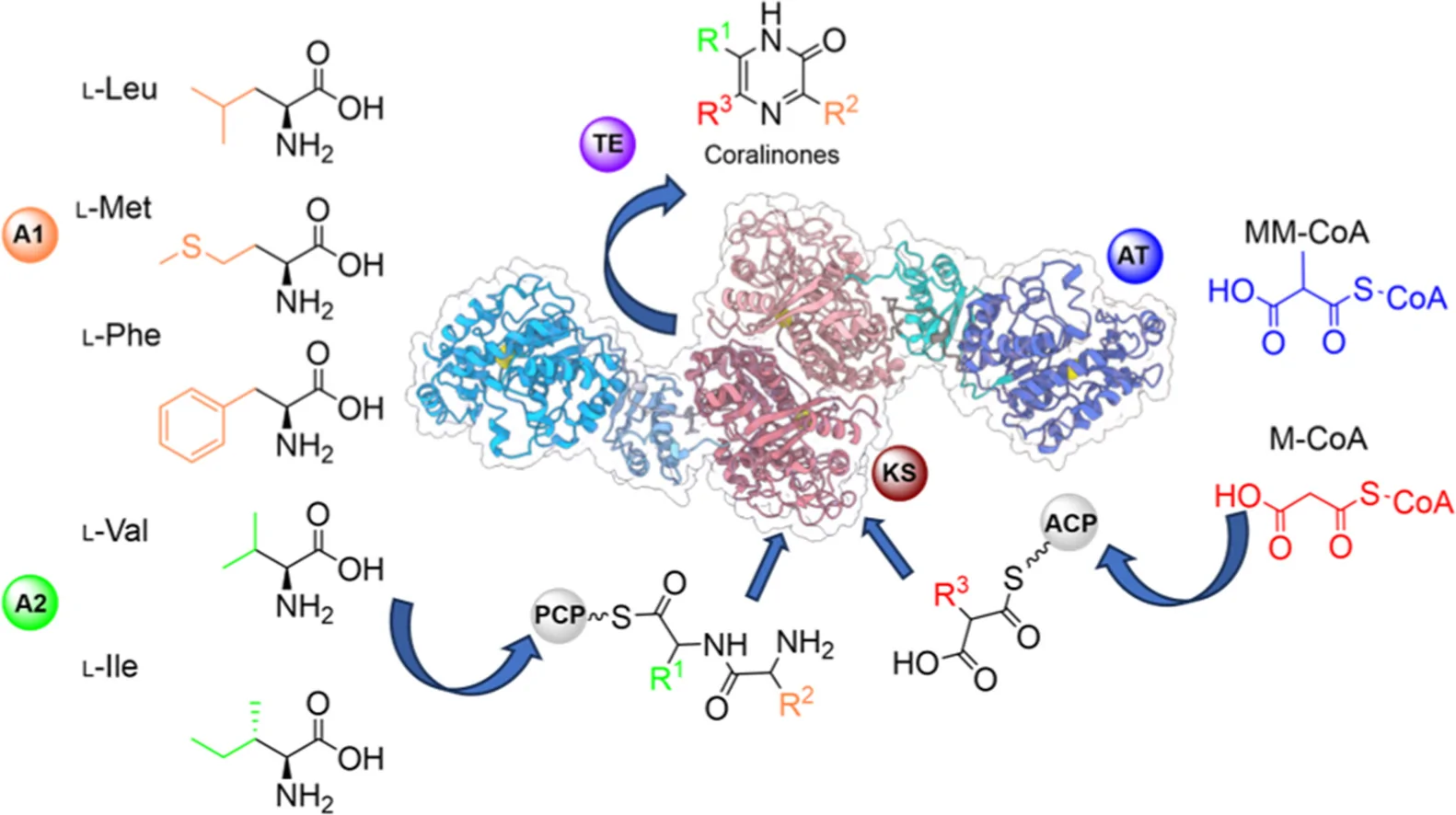

## 1 Introduction

Bacterial type I NRPS‑PKS hybrids, which employ a modular domain arrangement, are responsible for the biosynthesis of many hybrid molecules [1], including epothilone [2], rapamycin [3], bleomycin [4], and yersiniabactin [5]. Protein-protein interactions are necessary within NRPS‑PKS hybrid assembly lines to achieve accurate linkage between distinct biosynthetic intermediates [6]. Two architectures govern this process: tethered (modules covalently linked in one polypeptide) and separate (modules as distinct subunits). These architectures also differ in their communication strategies: separate systems use docking domains, whereas tethered systems rely on linkers and direct carrier protein-condensation contacts (Figure S1). Such interactions shape the effective substrate recognition by different domains [7]. Engineering these interaction interfaces can expand product diversity, but may also abolish overall biosynthetic activity [8]. Therefore, internal engineering targeting substrate recognition adenylation (A) domains and acyl transferase (AT) domains, as well as downstream condensation domains (C) and KS domains, constitutes a more robust strategy [9,10].

In the PKS module, the AT domains typically exhibit strict substrate specificity, as dictated by their conserved substrate recognition motifs [11,12]. Likewise, canonical KS domains act exclusively on simple acyl intermediates, whereas specialized KS domains in tethered-type hybrids can process peptidyl substrates, with substrate scope jointly modulated by pocket residues and CP-KS interfacial interactions [13]. While some studies have explored the substrate promiscuity of AT domains [14,15], investigations into the intrinsic substrate promiscuity of KS domains remain limited. Structural characterization of the broadly selective KS domain will provide key insights into the molecular basis of the enzyme’s substrate flexibility and may offer a strategy for guiding the engineering of other canonical KS domains.

CorA, a tethered-type NRPS‑PKS hybrid megasynthase from the myxobacterium *Corallococcus exiguus* SDU70, serves as an ideal model to investigate these pocket‑related principles in pyrazinone biosynthesis. Unlike previously characterized pyrazinone biosynthetic routes that depend entirely on NRPS assembly lines [16,17] (Figure S2a), CorA incorporates an additional PKS module to introduce a C5‑alkyl substituent, which distinguishes its products from those generated by pure NRPS pathways [18]. The assembly cascade begins with two NRPS A domains, which selectively recognize three and two classes of hydrophobic amino acids, respectively, to yield a dipeptidyl intermediate tethered to the terminal T domain. This T‑tethered intermediate is subsequently delivered to the downstream PKS module, where the C‑terminal KS‑AT‑ACP domains catalyze its condensation with either M‑CoA or MM‑CoA, generating a T‑tethered dipeptidyl β‑keto thioester. This β‑keto thioester intermediate requires no additional enzymatic processing and spontaneously undergoes a series of thioester hydrolysis, decarboxylation, intramolecular cyclization, and aerobic oxidation reactions to form the mature C5‑alkylated pyrazinone scaffold (Figure S2b).

Prior biochemical observations established the intrinsic substrate flexibility of CorA. Even so, the structural and functional origins of this promiscuity, especially within the KS domain that accommodates multiple amino acid‑derived peptidyl intermediates, remain largely undefined. Here, we investigate the pocket‑related structural and functional determinants underlying the substrate promiscuity of CorA by combining *in vitro* reconstitution, cryo‑electron microscopy (EM), site‑directed mutagenesis, and molecular dynamics (MD) simulations. We first showed that full-length CorA can assemble six distinct 5-alkylated pyrazinone products from six amino acid combinations and MM-CoA extender units, thereby expanding the known substrate scope of CorA-KS domain. We next determined the 3.1-Å cryo‑EM structure of the CorA KS-AT didomain, which provides a defined structural template for an AT domain that accommodates two distinct acyl‑CoA substrates. Notably, within the KS domain, we further identified key substrate‑recognition motifs that underpin its promiscuity toward multiple amino acid‑derived intermediates. Moreover, mutational and computational analyses correlated changes in catalytic activity with specific structural features, providing mechanistic insights into the catalysis of chemically diverse substrates and laying the groundwork for future enzyme engineering.

## 2 Materials and methods

### 2.1 Materials

Malonyl-and methylmalonyl-acyl-coenzyme A (M-CoA and MM-CoA, respectively) substrates were commercially sourced from Sigma-Aldrich (Shanghai, China). Amino acid raw materials were supplied by Sangon Biotech Co., Ltd. (Shanghai, China). Streptavidin affinity chromatography resins were purchased from Tiandi Renhe Biotechnology (Shanghai, China).

### 2.2 Cloning, expression, and purification of full-length CorA

The *corA* gene fragment was ligated into the pET28a(+) vector, and the resultant recombinant plasmid was transformed into *Escherichia coli* BAP1 cells. Pelleted bacterial cells were resuspended in lysis buffer (20 mM Tris, pH 7.5, 100 mM NaCl). After sonication on ice and centrifugation, the clarified lysate was loaded onto Ni-NTA resin. Imidazole-containing elution buffer (20 mM Tris, pH 7.5, 100 mM NaCl, 300 mM Imidazole) was used to retrieve crude CorA, which was further purified through Strep‑Tag II affinity chromatography. The eluted sample was loaded onto pre-equilibrated Strep-Tactin agarose, washed with five column volumes of Tris‑HCl wash buffer (100 mM Tris‑HCl, pH 8.0, 150 mM NaCl, 1 mM EDTA), and eluted upon the addition of 2.5 mM desthiobiotin. A Superdex 200 gel filtration column equilibrated with 20 mM Tris (pH 8.0) and 100 mM NaCl was applied for final purification. All purified protein fractions were combined, flash-frozen with liquid nitrogen, and stored long-term at −80°C in the matching Tris-NaCl buffer.

### 2.3 In-gel digestion and LC-MS/MS measurement

Target protein samples were separated via SDS-PAGE. The CorA band of interest was excised and cut into ∼2 × 2 mm pieces. These gel pieces were destained with 0.5 mL Buffer I (25 mM ammonium bicarbonate supplemented with 50% acetonitrile) until the visible pigment faded away, followed by vacuum centrifugation to achieve complete dryness. Subsequently, the gel pieces were immersed in 0.1 mL of a 10 mM dithiothreitol solution at 56°C for 1 h to reduce disulfide bonds. After this reduction step, the gel pieces were rinsed with acetonitrile. For cysteine carbamidomethylation, 100 μL of 55 mM iodoacetamide (IAA) solution was introduced into the mixture, and the mixture was incubated for 30 min at ambient temperature (25-30°C) under dark conditions. Residual IAA solution was discarded afterwards. The gel pieces were then sequentially washed twice using 25 mM NH₄HCO₃ and acetonitrile separately and vacuum-dried again. The desiccated gel fragments were subjected to tryptic digestion at a trypsin/protein mass ratio of 1:20 (w/w) at 37°C for 12 h. Enzymatic hydrolysis was quenched by adding 20 μL 0.1% trifluoroacetic acid. The extracted peptide mixtures were then desalted using C18 ZipTip pipette tips (Millipore, Billerica, MA, USA) prior to instrumental detection. Peptide separation was performed on an Ultimate 3000 chromatographic system (Dionex, Sunnyvale, CA, USA) connected to a Q Exactive mass spectrometer (Thermo Fisher Scientific, Waltham, MA, USA). The mobile phases consisted of solvent A (ultrapure water containing 0.1% formic acid) and solvent B (acetonitrile containing 0.1% formic acid), with a constant flow rate set at 0.4 μL·mL⁻¹. The linear elution gradient was programmed as follows: 5–8% solvent B within 0–6 min; 8–30% solvent B for 6–40 min; 30–60% solvent B from 40–45 min; 60–80% solvent B from 45–48 min; and finally maintained at 80% solvent B from 48–60 min.

### 2.4 Database searching

Raw tandem mass spectrometry (MS/MS) datasets were searched using Proteome Discoverer (Thermo Fisher Scientific, Version 1.4) against the in-house sequence database of CorA. The search parameters were configured as follows: trypsin was selected as the protease, permitting up to two missed cleavage sites. Cysteine carbamidomethylation was defined as a fixed modification, while methionine oxidation and serine pantetheinylation (Ppant, +326.10 Da) were assigned as variable modifications. Default precursor ion and fragment ion mass tolerances for Orbitrap acquisition were adopted throughout analysis. All peptide-spectrum matches were filtered to guarantee the reliable identification of CorA and accurate localization of Ppant modification sites.

### 2.5 Site-directed mutagenesis

The mutagenesis PCR reaction was performed using 50 ng of pET28a-CorA-WT as template and 0.2 μM of each forward/reverse primer pair (details of the primers employed for mutagenesis can be found in Table S1), and 10 μL of 2 × Phanta Max Master Mix (Vazyme Biotech Co., Ltd., Nanjing, China) in a total reaction volume of 25 μL. *Dpn*I digestion was performed with 1 μL of *Dpn*I (2 U·μL^–1^) per 25 μL reaction solution for 2 h at 37°C. Then, 2 μL of the digested mixture was used to transform 50 μL DH5α (approximately 5 × 10⁷ cells). The mutant plasmids were purified and subjected to sequencing at Sangon Biotech.

### 2.6 Kinetic analysis

The kinetic parameters of the enzyme were determined using a DTNB (5,5′-dithio-bis-2-nitrobenzoic acid)-based assay that monitors the release of free CoA. Since the standalone AT domain could not be expressed alone, all kinetic assays were performed using the recombinant KS-AT didomain protein. Reactions were carried out at room temperature in a 100 μL mixture containing 50 mM sodium phosphate buffer (pH 7.5), 1 μM KS-AT protein, 6.25–100 μM acyl‑CoA substrate, 0.2 mM DTNB, 1 mM EDTA, 1 mM TCEP, and 10% glycerol. The absorbance increase at 412 nm was continuously monitored using a microplate spectrophotometer. Kinetic parameters were calculated with a nonlinear regression fit to the Michaelis–Menten model using GraphPad Prism 8.3.0.

### 2.7 *In vitro* enzymatic assays and product detection

The systematic *in vitro* activity assay, including reaction formulation, negative control construction, sample pretreatment, and HPLC-HRMS detection gradient, was performed as comprehensively documented previously [18].

### 2.8 Cryo-EM data acquisition and processing

For cryo-EM, 3 μL CorA (2 mg·mL⁻¹) was loaded onto glow-discharged Quantifoil R1.2/1.3 gold grids (Quantifoil Micro Tools, Jena, Germany) and then blotted and plunge-frozen with a Vitrobot Mark IV (Thermo Fisher Scientific, Waltham, MA, USA). Cryo-EM datasets were collected on a 200 kV Glacios G2 microscope (Falcon 4i detector, EPU) (Thermo Fisher Scientific, Waltham, MA, USA) at 130,000 × magnification (0.89 Å pixel size, total dose 40 e⁻·Å⁻²). A total of 5,816 movies with defocus values from −2.5 to −1.0 μm were recorded, each split into 40 frames over an exposure of 3 s.

All single-particle processing steps were performed in CryoSPARC v4.6.2 [19]. Raw micrographs were motion-corrected using MotionCor2 [20] and subjected to CTF calculation using Gctf [21]; defective micrographs were removed manually. Approximately 4.85 million particles were auto-picked and subjected to iterative 2D classification with a 280-Å mask. Qualified averages were used to generate an *ab initio* 3D model, and subsequent 3D classification screened 58,128 dimer particles for non-uniform refinement under the same mask. C2 symmetry and serial CTF/defocus optimization yielded a 3.13-Å global reconstruction. Symmetry expansion and local refinement of the AT domain on the same particle subset produced a 3.60-Å local map. Resolutions were judged by gold-standard FSC = 0.143, and local resolution maps were visualized with Chimera X [22].

### 2.9 Model building

The AlphaFold3-predicted CorA structure served as the template for KS-AT didomain *de novo* modeling. The initial atomic model was docked into the cryo-EM density map via Phenix map_to_model [23]. Unstructured polypeptide segments were manually traced in Coot [24].

The full CorA-KS-AT model covers the KS (amino acids 1641–2069) and AT domains (amino acids 2163–2449), LD linker (amino acids 2070–2162), and PAL segment (amino acids 2450–2511). MolProbity [25] was used to validate stereochemical quality, protein interfaces were analyzed in ChimeraX, and structural illustrations were rendered using ChimeraX and PyMOL.

### 2.10 Preparation of protein–substrate complexes using docking models

AutoDock Tools 1.5.6 was used to handle the pretreatment steps for both the CorA protein and small-molecule substrates, including solvent removal, polar hydrogen addition and partial charge calculation, followed by formatting the output as a PDBQT file. Docking computations were run with AutoDock Vina 1.1.2 [26]. The best-scoring pose was used as the starting structure for subsequent MD simulations.

### 2.11 Computational simulations and molecular docking analysis

Enzymatic protonation states at pH 7.0 were predicted using the H++ web server [27]. All MD systems were built and computed with graphics processing unit (GPU)-accelerated Gromacs 2024 [28]. Protein residues were parameterized using the AMBER-ff19SB force field [29], while small-molecule ligands were structurally optimized and frequency-analyzed via ORCA 6.0.1 [30]. Ligand topological parameters were further processed with Multiwfn [31] and AmberTools. Water geometry and hydrogen-containing bonds were constrained using the SETTLE and LINCS algorithms, respectively, and long-range electrostatic interactions were calculated via the PME method. Finally, the gmx_MMPBSA module [32] was utilized to calculate the enzyme–ligand binding free energies from the MD trajectories.

## 3 Results and discussion

### 3.1 CorA exhibits broad substrate promiscuity *in vitro*

CorA, a tethered-type NRPS‑PKS hybrid megasynthase from the myxobacterium *C. exiguus* SDU70, is responsible for the production of 5-alkylated pyrazinones (Fig. 1a). We previously reconstituted the biosynthetic function of CorA *in vitro* and examined the substrate promiscuity of its A and AT domains [18]. In that study, to define the substrate specificity of the two A domains, we first performed a combinatorial screen in which CorA's cognate amino acids were paired with each of the remaining 19 canonical amino acids. UPLC analysis revealed that only six amino acid combinations: Leu-Val, Leu‑Ile, Met‑Ile, Met‑Val, Phe‑Ile, and Phe‑Val, could be utilized by CorA, yielding products **1–6** with M‑CoA serving as the extender unit (Figure S3a). Next, we separately expressed the A^1^ domain protein and characterized its substrate specificity using ATP/PP_i_ exchange assays; we extended this approach to A^2^ in the present study using an MBP-fused soluble construct (Figure S3b). The results confirmed that A¹ recognizes Leu, Met, and Phe, whereas A² is specific for Ile and Val, indicating that neither domain protein accepts any of the other canonical substrates tested. In that work, we also used a separately expressed PKS module to define the substrate promiscuity of the AT domain. With Leu‑Val‑SNAC as the substrate and MM‑CoA as the extender unit, the CorA‑PKS reaction yielded the corresponding 5‑ethylated product **7**, confirming that MM-CoA is accepted by the AT domain alongside M-CoA. Collectively, these findings indicate that the A and AT domains of CorA each display inherent substrate promiscuity.

**Fig. 1:**
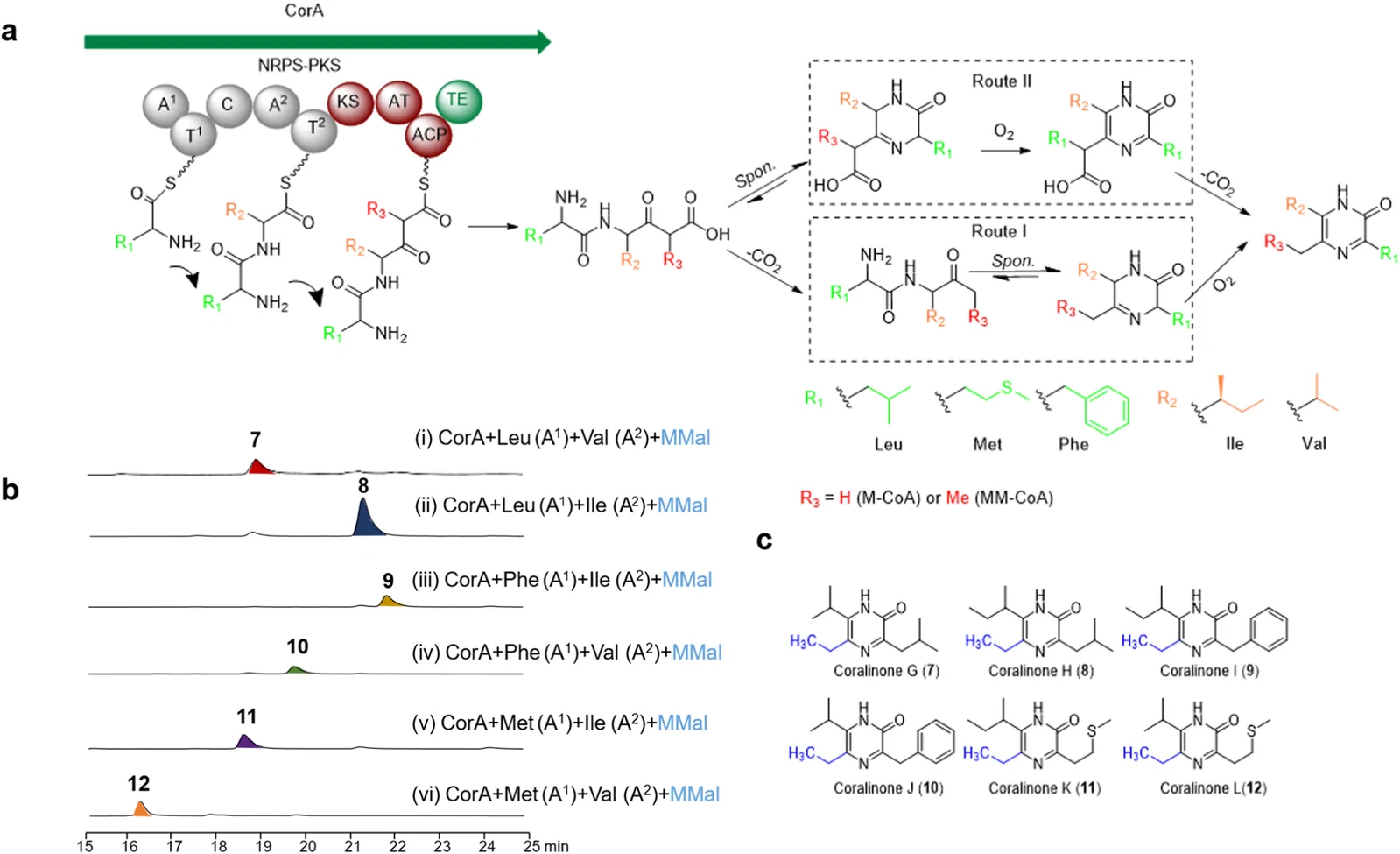
Functional reconstitution and substrate promiscuity of the CorA hybrid NRPS-PKS module. (a) Schematic illustration of the catalytic cascade of the hybrid NRPS-PKS megasynthetase CorA. Wavy linkages denote variable side chains: R_1_ corresponds to the side chains of Leu, Met, or Phe (loaded by domain A^1^); R_2_ corresponds to the side chains of Ile or Val (loaded by domain A^2^); R_3_ is derived from the two PKS extender units. (b) HPLC chromatograms of *in vitro* reactions showing the production of six distinct coralinone derivatives (peaks **8**–**12**) from different combinations of amino acid (Ile/Leu/Val/Phe/Met) and extender (MM-CoA) substrates. (c) Chemical structures of the six coralinone derivatives in (b).

To assess whether the inherent substrate flexibility of the individual A and AT domains operates cohesively within the full-length megasynthase, we reconstituted full-length CorA *in vitro* with the six pairwise amino acid combinations and MM-CoA extender units in this work. HPLC-DAD and LC-MS analyses revealed that, when MM‑CoA was supplied, full‑length CorA converted all six amino acid combinations into the corresponding 5‑ethylated pyrazinone products (**7**–**12**) (Figs. 1b, c, and S5). Together with the M‑CoA–derived products **1–6** described above, these data demonstrate that full-length CorA performs combinatorial catalysis with six dipeptide units and two acyl-CoA extender units, yielding 12 distinct pyrazinone products (Figure S4a).

This broad substrate accommodation is notable, considering that in modular NRPS‑PKS systems, substrate substitution frequently compromises productivity. Even if the upstream loading or condensation steps proceeded, later steps such as chain extension by the KS domain or product release may exhibit poor efficiency [8]. This finding suggests that the observed promiscuity of CorA is not confined to the A or AT domains, but reflects coordinated tolerance throughout the full assembly machinery, particularly for the downstream KS domain. Thus far, studies concerning the intrinsic substrate promiscuity of KS domains remain limited. Although several KS domains at NRPS‑PKS junctions have been shown to accommodate non‑canonical substrates: BaeJ‑KS2 domain tolerates configuration inversion [33]; ClbI‑KS domain accommodates aminomalonyl‑and aminoketone‑bearing building blocks [34]; HMWP1‑KS domain accepts cysteine‑derived intermediates [5]; and OzmQ‑KS1 domain recognizes a glycine‑derived β‑formamide intermediate [13]. However, these examples are restricted to adaptation toward particular substrates; none of these display the broad substrate tolerance observed for CorA‑KS domain. Therefore, structural characterization of CorA could yield new insights into its broad substrate promiscuity. In turn, these findings may inform future engineering of NRPS‑PKS assembly lines.

### 3.2 Cryo-EM structure of the KS-AT didomain of CorA-PKS

To establish a structural basis for the broad substrate tolerance of CorA, we further solved the cryo-EM structure of full-length CorA. After recombinant expression in *E. coli* BAP1 cells, the protein was purified to high homogeneity using sequential His-tag and Strep-tag affinity purification (Figure S6a). Size-exclusion chromatography (SEC) revealed a single symmetric peak with an apparent molecular weight of 424 kDa, which is substantially higher than the theoretical monomeric mass (310 kDa) but lower than the expected dimer (620 kDa). This discrepancy is likely due to a reversible monomer-dimer equilibrium in solution (Figure S6b). Given that multi-step purification and sample handling may cause domain dissociation or partial degradation, we further analyzed the purified CorA by MS. The peptide coverage exceeded 90% of the protein sequence, confirming the integrity of the full-length construct (Figure S7). This analysis also confirmed the presence of the covalently attached Ppant arm (Figure S8). Despite these efforts, iterative rounds of 2D classification and 3D refinement yielded clear density maps only for the KS-AT didomain, whereas the remaining regions of CorA, particularly the NRPS module, remained invisible (Figures S9a and S9c). These results reveal that the PKS core region features greater local structural order and conformational stability relative to other highly dynamic modules and carrier‑protein‑associated domains [35]. Although this local structure does not cover the entire CorA protein, it contains key catalytic regions essential for extender unit selection and chain elongation. Therefore, this KS-AT structure may help reveal the molecular basis underlying the product diversity of CorA.

The cryo-EM density map clearly resolved a dimeric core architecture with prominent two-fold rotational symmetry (Fig. 2b). This symmetry axis is oriented vertically in the top view and faces the viewer in the bottom view of Fig. 2b. In line with this, 2D class averages displayed distinct two-fold symmetric projections (Figure S9b), even without imposing symmetry during particle alignment and averaging. This demonstrates that the dimeric conformation is the predominant assembly state of CorA. Previous work on the PKS-NRPS hybrid HMWP reported the co-existence of monomeric and dimeric species in solution [36], indicating that KS-mediated dimerization does not fully lock the full-length enzyme in a dimeric state. In contrast, the PKS-NRPS hybrid ClbK is dimeric in solution, as determined by SEC and SAXS. However, its isolated Clb-PKS domain is monomeric in solution but dimeric in the crystal, with the dimer interface formed mainly through the KS-KS interface, resembling classical dimeric PKSs [37]. Taken together, our observations extend this emerging picture by demonstrating that CorA also undergoes reversible monomer-dimer equilibrium in solution. Transient dimeric assemblies alongside monomers account for its elevated apparent molecular weight by SEC, highlighting a conserved, KS-dependent dynamic dimerization mode shared by NRPS-PKS and PKS-NRPS hybrid enzymes.

**Fig. 2:**
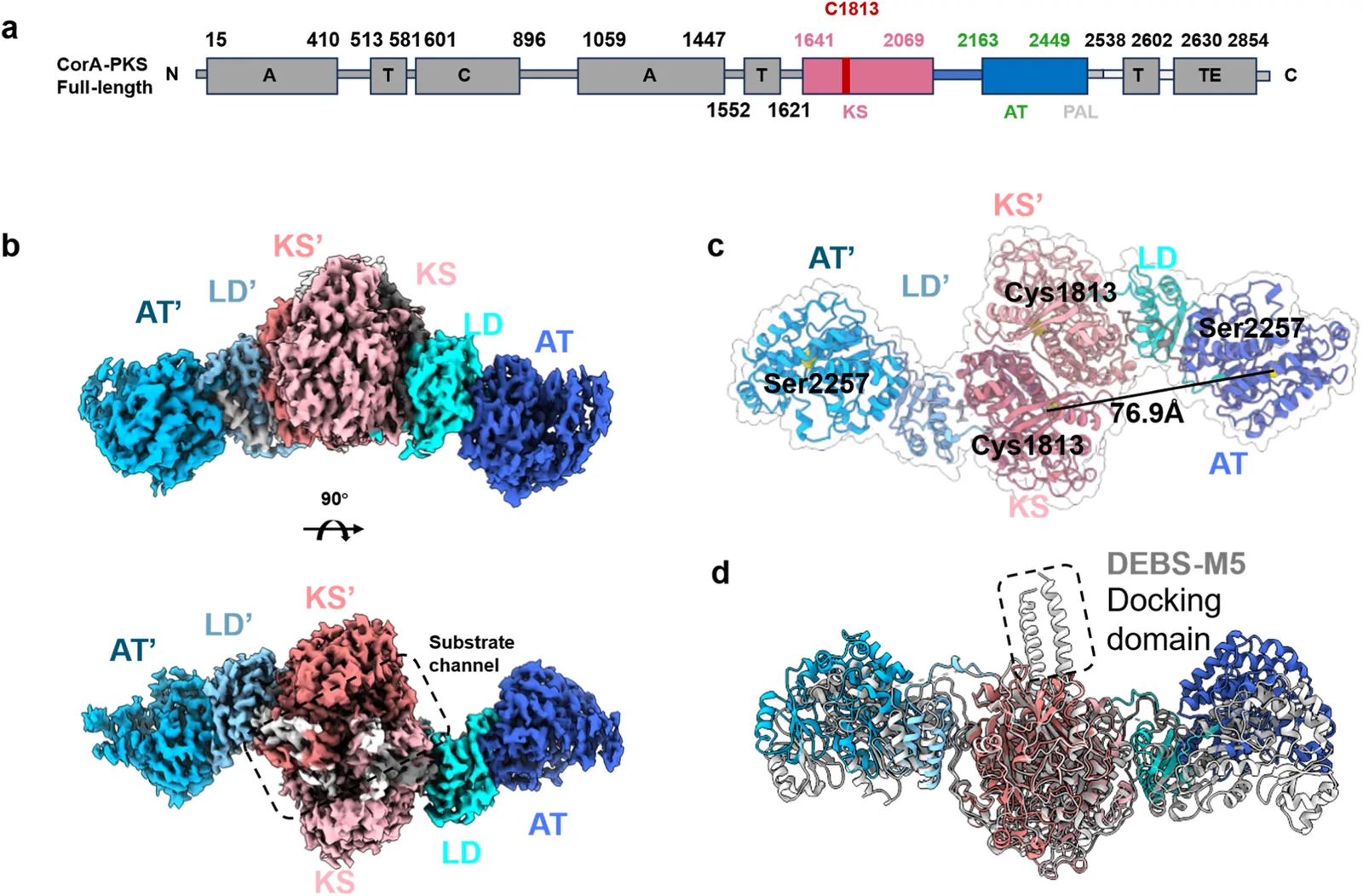
Domain architecture and cryo-EM structure of the CorA NRPS-PKS module. (a) Linear domain organization of full-length CorA-PKS, with unresolved regions marked. Catalytic Cys1813 in the KS domain is highlighted (unresolved regions are indicated in gray). (b) Cryo-EM structure of the CorA-KS-AT didomain dimer in two orthogonal views, showing the KS (pink), AT (blue), and linker/docking (LD, cyan) domains. The substrate channel is indicated in the bottom view. (c) Demonstration of the dimer interface formed by KS domain, highlighting the catalytic residues Cys1813 (KS) and Ser2257 (AT), with a 76.9 Å distance between the active sites. (d) Structural superposition of CorA-KS-AT (colored) with the canonical DEBS module 5 KS-AT structure (gray).

The final 3D reconstruction achieved an overall resolution of approximately 3.13 Å (Figure S9d), with varying local resolutions throughout the dimeric assembly (Figure S9d). The central KS dimer region was best resolved, reaching a local resolution of 2.8 Å (Figure S9g). In contrast, the intrinsically flexible AT domains were poorly resolved in the full-length dimeric map. Targeted local refinement with a tailored soft mask improved the AT domain density, yielding a high-quality local map at 3.6-Å resolution (Figures S9e and S9h). This high-quality cryo-EM density map enabled the unambiguous atomic modeling of the core domains. Representative well-resolved regions of the KS, LD, and AT domains are shown in Figure S10.

However, unlike typical *cis*-AT PKSs such as DEBS module 5, CorA-PKS lacks the helical elements that constitute docking domains [[38], [39], [40]] (Fig. 2d). This distinctive KS-AT arrangement is also consistent with that observed in ClbK, ClbC, and ClbI from the colibactin biosynthetic gene cluster [34,41] (Figure S11a), as well as the didomain core structure of *Mycobacterium tuberculosis* PKS13, a key enzyme in mycolic acid biosynthesis (Figure S11b). By contrast, this arrangement is distinct from the arch-like dimeric conformation of full-length pikromycin synthase module 5 (PikAIII), in which the two AT domains bend downward to form the supporting legs of the arch [42] (Figure S11c).

Structural analysis of the KS dimer interface using the PISA server [43], revealed that each monomer contributes a buried surface area of approximately 2644 Å² to the dimeric assembly. The interface is stabilized by numerous hydrophobic interactions, along with several salt bridges and approximately 28 interfacial hydrogen bonds (Figure S12). The active sites of the KS and AT domains are spatially separated, with their substrate-binding pockets opening in opposite directions. The catalytic cysteine (Cys1813) of the KS domain is located ∼76.9 Å from Ser2257, the active site residue of the nearest AT domain (Fig. 2c). The cavity leading to the catalytic cysteine (Cys1813) of the KS domain faces toward the AT domain of the opposing monomer (Figure S13a), whereas the entrance to the AT active site, centered on the catalytic serine (Ser2257), is directed downward (Figure S13b).

To further investigate the interactions involving NRPS-PKS, we also attempted to trap T^2^-KS and KS-ACP complexes. Unfortunately, soluble standalone recombinant T^2^ and ACP proteins could not be obtained. We therefore performed interaction predictions for the ACP-KS and T^2^-KS complexes using AlphaFold 3. A transacylation-state ACP-KS model was obtained (Figure S14a)—the ACP associates predominantly with the *trans* protomer of the KS homodimer while making no substantial contacts with the AT domain, consistent with published structures of type I modular PKSs [44]. In this predicted model, the ACP docks at the upper region of the KS substrate-channel opening, with interfacial contacts mediated by the αII and αIII helices and loop I [45]. The distance between the phosphopantetheine-anchoring serine of the ACP and the KS catalytic cysteine is within 15.6 Å, satisfying the spatial requirement for transacylation.

Given the high structural homology between the T and ACP domains, the AlphaFold 3 model of the T^2^-KS complex closely resembles that of the ACP-KS complex. However, this prediction may be compromised by the scarcity of experimentally determined T-KS structures, as only one such structure has been reported to date [34]. In this reported structure, an intra-module ACP and an inter-module T can occupy two non-overlapping binding surfaces on the KS within NRPS-PKS hybrid assembly lines: the intra-module ACP docks at the upper opening of the KS substrate channel, whereas the T binds to the lower-lateral region of the KS substrate channel (Figure S14b). Notably, beyond the helical elements of the T four-helix bundle, the C-terminal extended peptide of ClbH-T adopts a helical conformation. This motif forms a relatively small, buried interface at the dimeric groove of the KS, thereby mediating T recognition and subsequent downstream interactions (Figure S14b). We further predicted the monomeric structure of the CorA‑T^2^ using AlphaFold 3. The predicted structure reveals that CorA‑T^2^ lacks the C‑terminal helical element observed in ClbH‑T (Figure S14c). These predictions suggest that CorA‑T^2^ may engage with the KS in a mode distinct from that of the ACP-KS interaction, a hypothesis that awaits experimental validation.

### 3.3 Dual substrate-binding pocket of the CorA-AT domain

In line with structurally characterized AT domains, CorA-AT exhibits a canonical two-domain fold, comprising a large α/β-hydrolase core domain and a small ferredoxin-like domain (four parallel β-strands and two α-helices) (Figs. 3a and S15a). The catalytic residues are located in the interdomain cleft, as observed in other ATs: with Ser2257 within the conserved GHSLG motif (between α5 and α6) and His2356 in the substrate-specific motif (^2353^SGFH^2356^ on the β4–α10 loop) constituting a catalytic dyad (Figure S15b**)**. This conserved His-Ser dyad arrangement implies a catalytic mechanism analogous to that of other PKS AT domains.

**Fig. 3:**
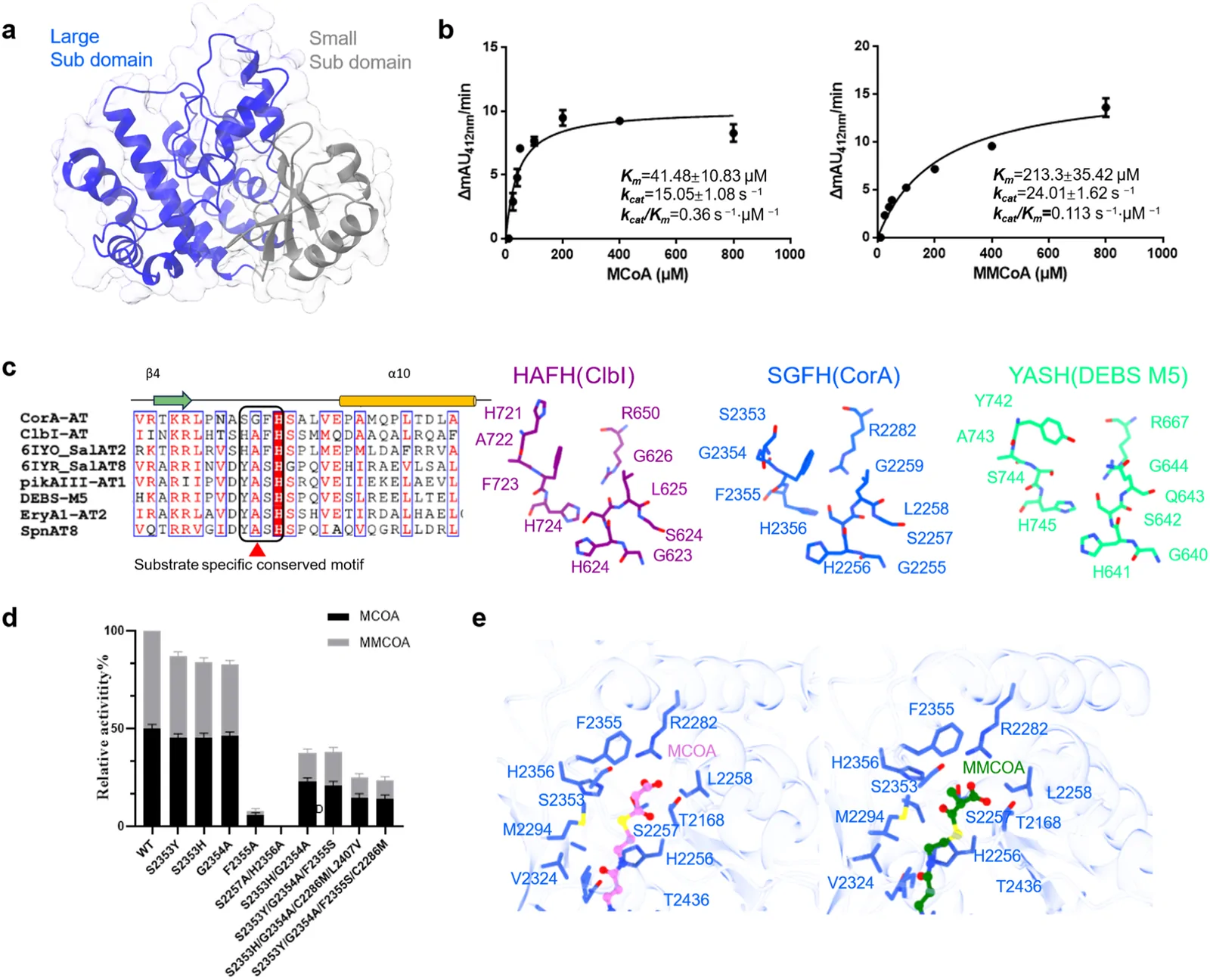
Structural and functional analysis of the CorA-AT domain. (a) Overall structure of the CorA-AT domain, showing the large (blue) and small (gray) subdomains. (b) Michaelis–Menten kinetic analysis of CorA-AT with M-CoA and MM-CoA. (c) Sequence alignment of AT domains highlighting the substrate-specific motif, with corresponding structural comparisons of the active site residues in CorA (SGFH), ClbI (HAFH), and DEBS M5 (YASH). (d) Relative activity of wild-type CorA and its mutants toward M-CoA and MM-CoA. All enzymatic assays were performed using recombinant full-length CorA proteins. (e) Docking models of the CorA-AT active site bound to M-CoA (pink) and MM-CoA (green), showing key interacting residues.

The AT domains in modular PKSs exhibit intrinsic specificity for several α-carboxyacyl-CoA substrates, with M-CoA and MM-CoA being the predominant choices, whereas ethylmalonyl-CoA (EM-CoA) is selected comparatively infrequently [46]. Substrate selectivity is governed by conserved active-site motifs, such as YASH for MM-CoA and HAFH for M-CoA, which have been identified through structure-based sequence alignments [47]. *In vitro* biochemical assays confirmed the inherent substrate promiscuity of CorA-AT, which utilizes both M-CoA and MM-CoA as extender units to generate distinct products. CorA-AT shows moderate specificity for its native substrate M-CoA (*k*_cat_*/K*_m_ = 0.4 s^−1^⋅μM^−1^; *k*_cat_ = 15.1 ± 1.1 s^−1^; *K*_m_ = 41.5 ± 10.8 μM), with a *k*_cat_*/K*_m_ value ∼3-fold higher than that of MM-CoA (Fig. 3b). Sequence alignment and structural analysis revealed that the substrate recognition motif of CorA-AT is an atypical SGFH sequence (Fig. 3c). To probe the contribution of this motif to substrate promiscuity, targeted site‑directed mutagenesis was performed using the full‑length CorA protein as the template. We mutated the non-canonical SGFH motif to the MM-CoA‑specific YASH motif and to the M-CoA‑specific HAFH motif, both individually and in combination, generating a series of variants including S2353H,/Y, G2354A, F2355S, H2356A, S2353H/G2354A, and S2353Y/G2354A/F2355S (Figure S16). Compared with the wild-type enzyme, only the single-point mutants S2353H/Y, and G2354A exhibited moderately reduced activity toward both M-CoA and MM-CoA, whereas the F2355S substitution caused drastic impairment of catalytic activity. Double substitutions at the catalytic dyad (S2257A/H2356A) completely abolished enzymatic activity. By contrast, the S2353H/G2354A double mutant and the S2353Y/G2354A/F2355S triple mutant retained approximately 50% of the activity of the wild-type enzyme. Notably, none of these variants displayed a significant shift in substrate selectivity (Fig. 3d), suggesting that residues outside the conserved SGFH motif also contribute to substrate recognition and binding.

It is well-established that additional residues within the active-site cavity can also modulate substrate preference [8]. Accordingly, combinatorial mutagenesis has been employed in several studies to remodel AT substrate specificity. As demonstrated by Zheng and coworkers, an AT domain with native dual specificity for M-CoA and MM-CoA was engineered into an M-CoA-selective variant through substrate-motif swapping and targeted mutagenesis of two active-site residues [48]. A similar combinatorial strategy was employed to reprogram the substrate specificity of the salinomycin AT8 domain (SalAT8) [49]. Guided by these findings, we constructed two independent quadruple mutants of CorA based on the substrate-motif swapping mutant: S2353H/G2354A/C2286M/L2407V and S2353H/G2354A/L2258V/N2351V (Figure S17). However, our results revealed that both quadruple mutants retained the ability to utilize both acyl-CoA substrates, failing to alter the substrate preference (Fig. 3d). Consistent with the notion that simple motif transplantation often fails across PKS AT homologs, these known hotspot mutations cannot be directly transferred to CorA‑AT. Precise and rational engineering of CorA-AT substrate specificity would require additional structural templates from highly specific ATs or high-resolution structures of AT-substrate complexes to guide rational mutagenesis [15].

To elucidate the structural determinants underlying the substrate promiscuity of CorA-AT and to rationalize the activity changes of the mutants, we performed MD simulations. M-CoA and MM-CoA were separately modeled into the active site of CorA-AT based on the crystal structure of the FabD-M-CoA complex (PDB: 2G2Z) [38]. Both resulting complexes were subjected to 100-ns MD simulations and exhibited highly similar overall energetic profiles, with comparable balances in favorable gas-phase (GGAS) and unfavorable solvation (GSOLV) energies (Figures S18a and S18b). Per-residue energy decomposition further revealed that Arg2282 contributed strong, conserved favorable binding energy for both substrates, serving as a core residue for substrate stabilization. Most of the other pocket residues individually contribute only weakly to substrate binding. Within the SGFH motif, Phe2355 resides at the bottom of the substrate‑binding pocket and is responsible for substrate positioning (Fig. 3e). Its mutation (F2355S) disrupted this anchoring, causing a sharp drop in enzymatic activity. Ser2353 contributes unfavorable binding energy toward both substrates, acting as a conformational constraint that restricts substrate orientation (Figures S19a and S19b). Gly2354, by contrast, does not interact directly with the substrates but maintains the backbone flexibility and loop topology. The single mutations S2353H and S2353Y merely relieved the constraint imposed by Ser2353, resulting in moderate activity loss. The S2353H/G2354A double mutant exhibited more severe activity reduction, arising from combined side‑chain perturbation and backbone rigidification. Notably, the triple mutant S2353Y/G2354A/F2355S retained approximately half of the activity of the wild-type enzyme, likely because the bulky aromatic side chain of S2353Y introduced steric hindrance that partially compensated for the loss of positioning and hydrophobic interactions caused by F2355S. Similarly, quadruple mutants showed reduced activity, possibly due to altered hydrophobicity within the binding pocket.

We additionally assessed EM‑CoA as an alternative extender unit. To this end, EM‑CoA was generated from ethylmalonic acid using the malonyl‑CoA synthetase MatB and then added to the CorA reaction [50]. However, no coralinone product was detectable, demonstrating that EM-CoA is not accepted by the CorA-AT domain (Figures S20a and S20b). Molecular docking revealed that EM-CoA binds to the AT pocket, yet its ethyl group clashes with Phe2355 and His2356. This displaces the substrate, weakens the hydrogen bond with Arg2282, and misaligns the thioester relative to Ser2353, precluding acyl transfer. Longer aliphatic side chains would likely face similar steric exclusion (Figure S20c).

These results demonstrate that the SGFH motif, together with its surrounding residues, collectively sustains the substrate promiscuity of CorA‑AT. The combined effects of these residues shape a tolerant active‑site architecture that enables CorA‑AT to recognize two distinct extender units, and this naturally promiscuous pocket thereby provides a functionally defined template for future AT engineering.

### 3.4 Structural basis of the substrate promiscuity of the CorA-KS domain

The KS domain adopts a canonical thiolase fold featuring a five-layered αβαβα core and contains a catalytic triad comprising Cys1813, His1949, and His1989 (Figs. 4a and S21). The principal structural differences among KS domains occur in the variable loops at the base of the KS domain, particularly the "clasping loop" and "active site cap" as defined by Lohman and colleagues [13].

**Fig. 4:**
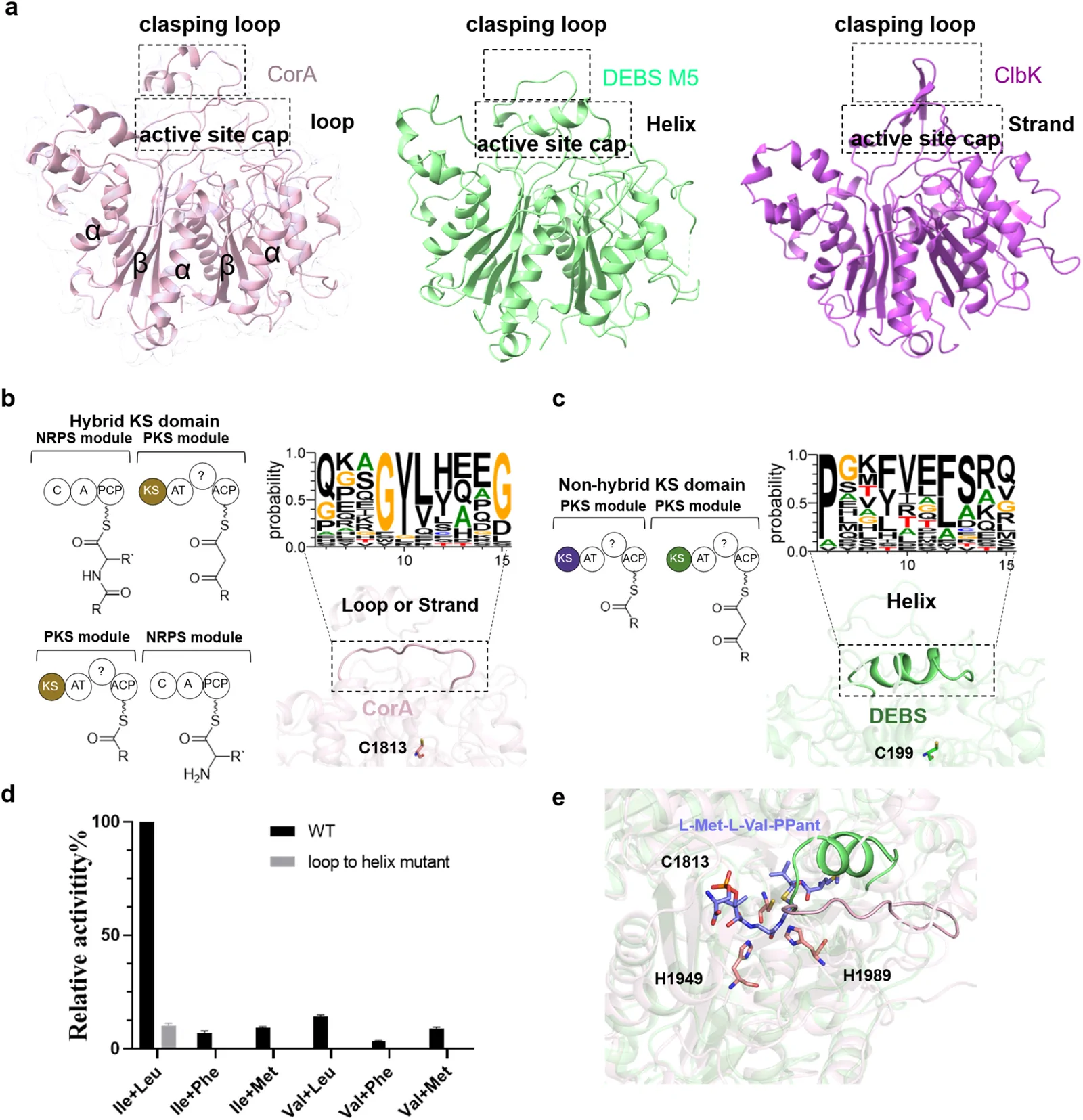
Structural and functional analysis of the active-site cap in hybrid and canonical KS domains. (a) Structural comparison of the active site cap and clasping loop in the CorA (pink, loop), DEBS M5 (green, helix), and ClbK (purple, strand) KS domains. (b) Consensus sequence logo and schematic of the cap region in KS domains of hybrid NRPS-PKSs, showing the loop/strand signature. (c) Consensus sequence logo and schematic of the cap region in KS domains of canonical PKSs, showing the conserved α-helix signature. (d) Relative activity of wild-type CorA and loop-to-helix mutant with different amino acid extender substrates. (e) Structural superimposition of the wild-type CorA-KS domain (pink) and DEBS-M5-KS domain (green). The L-Met-L-Val-Ppant substrate is shown as purple sticks docked into the active-site pocket of CorA.

Structural alignment of CorA-KS domain with the KS domain of DEBS module 5 revealed a largely similar overall fold [37]. In contrast, the α-helical element that serves as the active-site cap to enclose the substrate-binding pocket is conserved in canonical KS domains but is absent in CorA-KS domain, where it is replaced by a flexible loop (Fig. 4a). Notably, several type I PKS KS domains lacking this helical motif have been previously reported. This active-site cap adopts an α-helical conformation in DEBS module 5 KS, in BaeKS5 (a *trans*-AT PKS), and in other canonical *cis*-AT PKS KS domains, whereas it assumes a β8-strand fold in the KS domain of ClbC and other NRPS-PKS hybrid KS domains such as TAS1 [13,51] (Figs. 4a and S22). This non-α-helical cap fold may constitute a distinctive structural feature unique to NRPS-PKS hybrid enzymes. Distinct configurations of the active-site cap lead to pronounced differences in the overall geometry of the substrate-binding cavities. Although no obvious differences were observed in the peripheral Ppant-binding outer pockets among KS domain homologs [52], remarkable differences were found in the spatial volume and orientation of the inner acyl-binding pockets. Specifically, the inner acyl pockets of CorA-KS domain and other NRPS-PKS hybrid KS domains are substantially more spacious and open than those of canonical type I PKSs (Figure S23).

A phylogenetic tree constructed from functionally characterized amino acid-accepting KS domains revealed that the hybrid KS domain members form an independent evolutionary clade, clearly distinct from canonical type I *cis*-AT PKSs and *trans*-AT PKSs (Figure S24). Sequence conservation analysis of the active-site cap region was then performed for the KS domains in hybrid NRPS-PKSs and in *cis*-AT PKSs. As shown in the sequence logos, the α-helical cap of canonical KS domains displays high sequence conservation, with a dominant Pro at the N-terminus and conserved hydrophobic residues (Phe, Val, and Leu) throughout the helix (Figs. 4b and c). This conservation pattern reflects structural constraints imposed by the need to maintain the helical fold, as well as its functional role in narrowing the substrate-binding pocket. In contrast, the non–α-helical cap region in CorA-like hybrid KS domains lacks such a conserved helical signature. Instead, the corresponding loop sequence exhibits markedly higher variability, being enriched in small polar residues (Ser/Thr) and Gly that are partially conserved and likely facilitate the expansion of the inner acyl pocket.

We then docked six dipeptidyl-Ppant substrates into the CorA-KS domain active site, using the structure of the FabB-cerulenin complex (PDB: 8SMS) [53] as a template. The docking results revealed that all six substrates were fully accommodated within the binding pocket enclosed by the non–α-helical loop cap of CorA-KS domain. In contrast, structural superposition with the KS domain of DEBS module 5 showed that the corresponding α-helical cap would sterically clash with and occlude the extension of these bulkier substrates (Figs. 4e and S25). To further investigate the role of this non-α-helical loop cap in conferring broad tolerance to amino acid-derived substrates, we performed a loop-swap experiment in which the CorA-KS domain cap loop was replaced with the corresponding α-helical cap sequence from DEBS module 5. The resulting chimeric KS domain variant was assayed *in vitro* with six pairwise amino acid combinations using M-CoA as the extender unit. The chimeric enzyme produced only trace amounts of product **2** (Ile-Leu-M-CoA), whereas the formation of the other five products was completely abolished (Fig. 4d). These data demonstrate that the loop architecture of the CorA-KS domain cap is essential for accommodating bulky amino acid-derived substrates. Its replacement with the canonical α-helical cap (Met229-Gly242 from DEBS-M5) severely compromises the substrate promiscuity of the enzyme. Collectively, these findings highlight how sequence variation in the cap region has evolved to accommodate the specific substrates of NRPS-PKS hybrid systems.

## 4 Conclusions

This study demonstrated notable substrate promiscuity of the hybrid NRPS‑PKS enzyme CorA through the production of twelve distinct C5‑alkylated pyrazinones *in vitro*. It also revealed that substrate tolerance is inherent not only to the A and AT domains, but also to the KS domain, which displayed unusually broad substrate tolerance toward upstream intermediates.

While our cryo‑EM analysis was limited to the KS‑AT didomain, the structure we obtained nonetheless offers valuable structural insights into the substrate promiscuity of the AT and KS domains. Further biochemical, structural, mutagenesis, and MD analyses revealed that the atypical SGFH motif in the CorA-AT domain (Ser2353, Gly2354, and Phe2355), together with the surrounding residues, forms a permissive active-site pocket that accommodates both M-CoA and MM-CoA, enabling dual extender recognition. Single-point mutations were not enough to eliminate this broad substrate tolerance. Notably, the CorA‑KS domain adopts a non–α‑helical loop cap at the entrance of its active site, in contrast to the conserved α‑helical cap found in KS domains of canonical PKSs. Structural comparisons, phylogenetic analyses, and domain‑swapping experiments demonstrate that this loop cap forms a spacious substrate‑binding pocket that accommodates diverse bulky amino acid‑derived intermediates, establishing the non‑helical cap as a key structural determinant of both KS domain function in hybrid NRPS‑PKS modules and substrate promiscuity.

Taken together, our biochemical and structural analyses have identified two PKS domains that exhibit intrinsic substrate promiscuity. These results deepen our mechanistic understanding of hybrid biosynthetic enzymes and provide guidance for the future engineering of modular NRPS‑PKS pathways to expand structural diversity in natural products.

## Data availability statement

All discussed data are included in the manuscript or in the Supplementary Information.

The atomic coordinates of the CorA KS-AT di-domain have been deposited in the PDB (https://www.rcsb.org) under accession code 000027DN. The full cryo-EM density map of the KS-AT di-domain is available in the EMDB as EMD-81083, and the focused density map of the standalone AT domain has been assigned the accession code EMD-81084.

## CRediT authorship contribution statement

**Qingyu Yang:** Writing – original draft, Validation, Investigation, Data curation. **Qian Wang:** Validation, Data curation. **Xiaoju Li:** Validation, Data curation. **Moli Sang:** Validation, Conceptualization. **Tiandong Hu:** Validation, Conceptualization. **Degao Wang:** Validation, Conceptualization. **Lele Zhu:** Validation, Conceptualization. **Dalei Wu:** Supervision. **Yuezhong Li:** Supervision. **Shengying Li:** Supervision. **Changsheng Wu:** Supervision, Resources. **Hang Zhang:** Writing – review & editing, Validation, Supervision, Methodology, Investigation, Data curation, Conceptualization. **Wei Zhang:** Writing – review & editing, Writing – original draft, Validation, Supervision, Project administration, Methodology, Investigation, Funding acquisition, Data curation, Conceptualization.

## Declaration of competing interest

The authors declare the following financial interests/personal relationships which may be considered as potential competing interests: Given their roles as Executive Editor and Editorial Board Member, respectively, Dr. Shengying Li and Dr. Yuezhong Li had no involvement in the peer review of this article and had no access to information regarding its peer review. Full responsibility for the editorial process for this article was delegated to Dr. Jiazhang Lian.

## Appendix Supplementary materials

Image, application 1
