# Supplementary material for: Insights into pyrazinone biosynthesis from biochemical and cryo-EM studies of the hybrid NRPS-PKS enzyme CorA
Source: Eng Microbiol. 2026 Oct 8;6(4):100308. doi: 10.1016/j.engmic.2026.100308 (PMC13691573; doi:10.1016/j.engmic.2026.100308)
Supplement: Supplementary file 1 [file mmc1.docx]

**Supplementary Information**

**Insights into Pyrazinone Biosynthesis from Biochemical and Cryo-EM Studies of the Hybrid NRPS-PKS Enzyme CorA**

Qingyu Yang^[a] [b]^, Qian Wang^[b]^, Xiaoju Li^[a]^, Moli Sang^[b]^, Tiandong Hu^[a] [b]^, Degao Wang^[a]^, Lele Zhu^[a]^, Dalei Wu^[a]^, Yuezhong Li^[a]^, Shengying Li^[a]^, Changsheng Wu^[a]^, Hang Zhang^[a] [e]*^, Wei Zhang^[a] [b] [c] [d]*^

[a]	State Key Laboratory of Microbial Technology, Shandong University, Qingdao, Shandong 266237, China

[b]	Laboratory of Experimental Marine Biology, Institute of Oceanology, Chinese Academy of Sciences, Qingdao 266000, China

[c]	Laboratory for Marine Biology and Biotechnology, Qingdao Marine Science and Technology Center, Qingdao, Shandong 266237, China

[d]	University of Chinese Academy of Science, Beijing 100049, China

[e]	Shandong Provincial Key Laboratory of Deep Sea Bioresources Exploration and Utilization, Shandong University, Qingdao 266237, China

**Contents**

[Supplementary Tables	3](#_Toc239258040)

[Table S1. Primers used in this study.	3](#_Toc239258041)

[Table S2. Cryo-EM data collection, processing, model building, refinement and structural validation statistics.	4](#_Toc239258042)

[Supplementary Figure	5](#_Toc239258043)

[Fig. S1. Domain organization of PKS-NRPS hybrids	5](#_Toc239258044)

[Fig. S2. Biosynthetic pathways for pyrazinone natural products	6](#_Toc239258045)

[Fig. S3. Substrate selectivity of the CorA A₁ and A₂ adenylation domains.	7](#_Toc239258046)

[Fig. S4. Substrate promiscuity of CorA and characterization of diverse pyrazinone products	8](#_Toc239258047)

[Fig. S5. The HRESIMS spectra of the compounds in the positive ion mode in this study.	9](#_Toc239258048)

[Fig. S6. Estimation of purity and SEC analysis of CorA.	10](#_Toc239258049)

[Fig. S7. Mass spectrometry-based sequence coverage of CorA.	11](#_Toc239258050)

[Fig. S8. MALDI-TOF/TOF verification of phosphopantetheine (PPant) arm installation	12](#_Toc239258051)

[Fig. S9. Cryo-EM data processing and map reconstruction of CorA, related to Figure 2	13](#_Toc239258052)

[Fig. S10. Electron densities of selected secondary elements	14](#_Toc239258053)

[Fig. S11. Structural alignment of CorA-KS-AT and other ATs	15](#_Toc239258054)

[Fig. S12. Interaction interface between two CorA-KS-AT monomers.	16](#_Toc239258055)

[Fig. S13. Overall structure of the CorA-KS-AT homodimer.	16](#_Toc239258056)

[Fig. S14. AlphaFold3-predicted models of CorA carrier proteins and complexes..	17](#_Toc239258057)

[Fig. S15. Structure of the CorA-AT domain	18](#_Toc239258058)

[Fig. S16. SDS-PAGE analysis of purified CorA variants.	19](#_Toc239258059)

[Fig. S17. Structural overlay and in vitro functional assays of CorA-AT mutants.	20](#_Toc239258060)

[Fig. S18. Decomposed binding free energy components of CorA-AT with substrates.	21](#_Toc239258061)

[Fig. S19. Residue-level binding free energy contributions in CorA-AT complexes..	22](#_Toc239258062)

[Fig. S20. Substrate selectivity analysis of Cor AT.	23](#_Toc239258063)

[Fig. S21. CorA KS domain active site and key functional residues.	24](#_Toc239258064)

[Fig. S22. Structural classification of the active site cap in KS domains	25](#_Toc239258065)

[Fig. S23. Structural comparison of substrate-binding and ACP interaction modes.	26](#_Toc239258066)

[Fig. S24. Phylogenetic classification of KS domains	27](#_Toc239258067)

[Fig. S25. Docking models of CorA KS domain with diverse acyl-substrate intermediates	28](#_Toc239258068)

Supplementary Tables

**Table S1.** Primers used in this study.

| Primer | Sequence (5' to 3') |
| --- | --- |
| H4-A-F | gcgtccggattcgcgtccgcgctcgtggagcccgcgatgcagccgctcacgga |
| H4-A-R | ggctccacgagcgcggacgcgaatccggacgcgttcggcagccgcttcgtcctc |
| F3-A-F | gcgtccggagcgcactccgcgctcgtggagcccgcgatgcagccgctcacgga |
| F3-A-R | ggctccacgagcgcggagtgcgctccggacgcgttcggcagccgcttcgtcctc |
| S1-H-F | cgcacggcttccactccgcgctcgtggagcccgcgatgcagccgctcacggacc |
| S1-H-R | ggctccacgagcgcggagtggaagccgtgcgcgttcggcagccgcttcgtcctc |
| HAFH-F | gcgcacgcgttccactccgcgctcgtggagcccgcgatgcagccgctcacgga |
| HAFH-R | ggctccacgagcgcggagtggaacgcgtgcgcgttcggcagccgcttcgtcctc |
| G2-A-F | gcgtccgccttccactccgcgctcgtggagcccgcgatgcagccgctcacgga |
| G2-A-R | ggctccacgagcgcggagtggaaggcggacgcgttcggcagccgcttcgtcctc |
| S1-Y-F | gcgtacggcttccactccgcgctcgtggagcccgcgatgcagccgctcacgga |
| S1-Y-R | ggctccacgagcgcggagtggaagccgtacgcgttcggcagccgcttcgtcctc |
| F3-Y-F | gcgtccggataccactccgcgctcgtggagcccgcgatgcagccgctcacgga |
| F3-Y-R | ggctccacgagcgcggagtggtatccggacgcgttcggcagccgcttcgtcctc |
| YASH-F | gcgtacgcatcccactccgcgctcgtggagcccgcgatgcagccgctcacgga |
| YASH-R | ggctccacgagcgcggagtgggatgcgtacgcgttcggcagccgcttcgtcctc |
| Loop-alter-F | AGCCCGGGCGCGTTTACCGAATTTCGCAGCCAGGGCatcacctcgccggatgggcac |
| Loop-alter-R | GCTGCGAAATTCGGTAAACGCGCCCGGGCTGCTCATgagggagatgcctcccgccagg |

**Table S2.** Cryo-EM data collection, processing, model building, refinement and structural validation statistics.

|  | CorA NRPS-PKS (EMD- 81083, PDB 000027DN ) | CorA NRPS-PKS focused on AT domain (EMD-81084) |
| --- | --- | --- |
| Data collection and processing   Magnification   Voltage (kV)   Electron exposure (e^–^ /Å^2^)   Defocus range (μm)   Pixel size (Å)   Symmetry imposed   Initial particle images (no.)   Final particle images (no.)   Map resolution (Å)    FSC threshold   Map resolution range (Å)  Refinement   Initial model used   Model resolution (Å)    FSC threshold   Model resolution range (Å)   Map sharpening B factor (Å^2^)   Model composition    Nonhydrogen atoms    Protein residues    Ligands  B factors (Å^2^)   Protein   Ligand  R.m.s. deviations   Bond lengths (Å)   Bond angles (°)  Validation   MolProbity score   Clashscore   Poor rotamers (%)  Ramachandran plot   Favored (%)   Allowed (%)   Disallowed (%) | 130,000  200  40  -1.0 to -2.5  0.89  C2  4,845,167  58,128  3.13  0.143  1.9 – 10.2  Predicted by AlphaFold3  3.27  0.5  3.0 – 4.7  -131.4  13,082  1746  0  54.38  N/A  0.005  0.682  1.90  8.86  0.38  93.51  6.26  0.23 | 130,000  200  40  -1.0 to -2.5  0.89  C1  4,845,167  116,256  3.60  0.143  1.9 – 20.5 |

Supplementary Figure

Fig. S1. Domain organization of PKS-NRPS hybrids [1]. (A) Tethered hybrid type and separate hybrid type. (B) PKS‑NRPS hybrids containing tethered hybrid interfaces, (C) PKS-NRPS hybrids containing separate hybrid interfaces. Docking domains are represented as small circles.

Fig. S2. Biosynthetic pathways for pyrazinone natural products. (a) NRPS machineries for the biosynthesis of tryvalin, phevalin and aureusimines [2,3]. (b) NRPS-PKS hybrid assembly line CorA generates diverse C5-substituted pyrazinone scaffolds.

**Fig. S3.** Substrate selectivity of the CorA A^1^ and A^2^ adenylation domains. (a) UPLC profiling of dipeptide formation with three fixed residues (Ile, Val, Leu) paired against a panel of 19 amino acids. (b) ATP-PPᵢ exchange assays showing amino acid substrate preference of A^₁^ and A^₂^ domains. SDS-PAGE confirms protein purity of the recombinant A^1^ and MBP-A^2^ proteins.

**Fig. S4.** Substrate promiscuity of CorA and characterization of diverse pyrazinone products.(a) LC-MS chromatograms of in *vitro* CorA reactions reconstituted with distinct amino acid combinations, malonyl-CoA (M-CoA) or methylmalonyl-CoA (MM-CoA). Twelve coralinone derivatives (1–12) were produced.(b) Chemical structures of twelve pyrazinone products, coralinone A–L..

**Fig. S5** The HRESIMS spectra of the compounds in the positive ion mode in this study.

**Fig. S6.** (a) Estimation of purity and SEC analysis of CorA. Purification workflow and purity analysis of CorA protein, (b) CorA exists in dynamic monomer–dimer equilibrium in solution; SEC analysis gives an apparent molecular mass M_app_=424 kDa , compared with the calculated monomeric mass M_calc_=310 kDa.

**Fig. S7.** Mass spectrometry-based sequence coverage of CorA. MALDI-TOF/TOF MS analysis revealed >90% primary sequence coverage of SEC-purified CorA (Red: peptides matched by MS; gray: uncovered sequence regions).

**Fig. S8.** MALDI-TOF/TOF verification of phosphopantetheine (PPant) arm installation on the recombinant CorA domain (T1, T2, ACP).

**Fig. S9.** Cryo-EM data processing and map reconstruction of CorA, related to Figure 2. (a) A representative cryo-EM micrograph. Scale bar, 70 nm. (b) Representative 2D classes. (c) A flow chart for cryo-EM data analysis. (d)(e) The FSC (Fourier Shell Correlation) curves of CorA NRPS-PKS map (d) and focused local refined map of CorA AT domain (e). (f) Azimuth view of angle distributions of particles used for final reconstruction. (g)(h) Local resolution estimation of CorA NRPS-PKS map (g) and focused local refined map of CorA AT domain (h).

**Fig. S10.** Electron densities of selected secondary elements in the KS, LD and AT domains of CorA-PKS. The numbers in parentheses represent the range of the amino acid sequence.

**Fig. S11.** (a) Structural alignment of CorA-KS-AT and ClbK (in green, PDB 8XJU), ClbC (in purple, PDB 8XBL), ClbI (in red, PDB 8CJH). (b) Structural alignment of CorA-KS-AT and PKS13 (in gray, PDB 8CV1). (c) CorA-KS-AT structure fits into the cryo-EM density for PikAIII (in gray surface, EMD-5653).

**Fig. S12.** Interaction interface between two CorA-KS-AT monomers. The open-book view of the dimeric interface of the CorA-KS-AT. The residues involved in contact, including hydrogen bond, and salt bridge are analyzed using ePISA, and colored as indicated.

**Fig. S13.** Overall structure of the CorA-KS-AT homodimer. (a) view of the dimer down the twofold rotation axis with active site cavities of the KS domains (Cys 1813, yellow) indicated by dashed lines. In this orientation, the active site cavities for the AT domains are oriented away from the viewer. (b) active site cavity of the AT domain (Ser 2257, yellow , dashed oval) opening towards the viewer.

**Fig. S14.** AlphaFold3-predicted structural models of CorA carrier proteins and complexes. (a) Predicted structural models of CorA ACP and KS‑AT‑ACP (2:1 stoichiometry) complex. (b) Superposition of ClbH‑T and ClbC‑ACP complex structures (PDB: 8XJZ, 8XJT). (c) Side‑by‑side comparison of CorA‑T and ClbH‑T models, illustrating their C‑terminal extended peptides.

**Fig. S15.** Structure of the CorA-AT domain. (a) Overall fold of CorA-AT, with the large subdomain (blue) and small subdomain (gray). The substrate-binding pocket is highlighted. (b) Stereoview of the active site, showing key residues lining the pocket.

**Fig. S16.** SDS-PAGE analysis of purified CorA variants. Left panel: Coomassie blue-stained SDS-PAGE gel showing the purified CorA mutants (S2353Y, S2353H, G2354A, F2355A, S2357A/H2356A, S2353H/G2354A, S2353Y/G2354A, and Loop-helix-mutant). Middle panel: the purified CorA mutants (S2353H/G2354A/C2286M/L2407V, S2353H/G2354A/L2258V/N2351V) All proteins exhibited high purity and migrated at the expected molecular weight. Right panel: SDS-PAGE analysis of the CorA-KS-AT construct. Protein molecular weight markers (in kDa) are indicated on the left of each gel.

**Fig. S17.** Structural overlay and in vitro functional assays of CorA-AT mutants. Top panels show wild type SalAT8 MM CoA and SpnAT8 MM CoA models with previously reported mutagenesis sites annotated. Bottom panels show structural overlays of wild type CorA AT homology model, highlighting residues designed for quadruple mutagenesis.

**Fig. S18.** Decomposed binding free energy components of CorA-AT with (a) MCoA and (b) MMCoA. In this study, GGAS represents the total gas-phase energy, which consists of van der Waals energy (VDWAALS) and electrostatic energy (EEL); GSOLV denotes the total solvation energy, including polar solvation energy (EPB) and nonpolar solvation energy (ENPOLAR); TOTAL, total binding free energy. Error bars represent standard deviations.

**Fig. S19.** Residue-level binding free energy contributions in CorA-AT complexes. (a) MCoA-bound state; (b) MMCoA-bound state. The y-axis shows the decomposed binding energy (kcal/mol) per residue, where negative values indicate favorable contributions to ligand binding. Error bars represent standard deviations from simulation replicates.

**Fig. S20.** Substrate selectivity analysis of Cor AT. (a) HPLC‑MS profiles of in vitro MatB‑CorA coupled reactions. (b) Chemical structures of coralinone derivatives. (c) Docking comparison of MM‑CoA (magenta) and EM‑CoA (orange) within the Cor‑AT binding pocket.

**Fig. S21.** CorA KS domain active site and key functional residues. The overall fold of the CorA KS domain is shown in light pink cartoon representation. The dashed box highlights the conserved active site region, which is further magnified in the stereo view (inset). Key residues lining the substrate-binding pocket are displayed as sticks, including the canonical catalytic triad.

**Fig. S22.** Structural classification of the active site cap in KS domains from hybrid and canonical biosynthetic systems. KS domains are grouped into three classes based on the structural architecture of their active site cap: (1) non-hybrid KS domains (top, cyan/green/blue) from canonical PKS systems, which feature an α-helical cap; (2) tethered hybrid KS domains (middle, purple/yellow/pink) from NRPS-PKS hybrid systems, with a β-strand/loop cap; and (3) separate hybrid KS domains (bottom, orange/red) from NRPS-PKS hybrid systems, which lack the helical cap and contain only a flexible loop. Representative structures from each class are shown with their corresponding PDB IDs, highlighting the distinct cap architectures that distinguish canonical and hybrid KS domains.

**Fig. S23.** Structural comparison of substrate-binding and ACP interaction modes. (a) Overall structure of the FAS KS-AT didomain (PDB: 8SMS) in complex with cerulenin and two ACP domains (yellow). (b) Structural superposition of FAS (cyan) with canonical DEBS KS-AT (green) and CorA KS-AT (pink), highlighting the relative orientations of the KS domains and their bound substrates (sticks). (c) Surface representation of the substrate-binding pockets in CorA KS-AT (pink) and DEBS KS-AT (green), revealing differences in pocket volume and architecture.

**Fig. S24.** Phylogenetic classification of KS domains from hybrid and canonical biosynthetic systems. (left) the neighbor-joining phylogenetic tree of representative KS domains, showing three distinct clades: hybrid NRPS-PKS-associated KS domains (brown), canonical *cis*-AT PKS KS domains (purple), and non-amino acid-processing KS domains derived from hybrid NRPS-PKS systems (green). Hybrid KS domains, including CorA and homologs from OzmQ, ClbK, and TAS1, form a well-separated monophyletic group. (right) Schematic representation of the module contexts: hybrid NRPS-PKS and PKS-NRPS architectures, as well as canonical PKS-only modules within hybrid systems. This classification highlights the evolutionary and functional divergence between KS domains that interact with amino acid-derived substrates and those that act on pure polyketide chains in the same hybrid biosynthetic pathway.

**Fig. S25.** Docking models of CorA KS domain with diverse acyl-substrate intermediates. Each panel displays the modeled binding poses of distinct acyl-substrate intermediates (sticks, colored by atom type) within the CorA KS domain binding pocket (light pink cartoon). The corresponding chemical structures of the substrates are shown below each panel, illustrating the molecular basis for the broad substrate tolerance of the hybrid NRPS-PKS KS domain.

**REFERENCES**

[1] A. Miyanaga, F. Kudo, T. Eguchi, Protein-protein interactions in polyketide synthase-nonribosomal peptide synthetase hybrid assembly lines, Nat. Prod. Rep. 35 (2018) 1185-1209. DOI: 10.1039/c8np00022k.

[2] M. A. Wyatt, W. Wang, C. M. Roux, F. C. Beasley, D. E. Heinrichs, P. M. Dunman, N. A. Magarvey, Staphylococcus aureus nonribosomal peptide secondary metabolites regulate virulence, Science 329 (2010) 294-296. DOI: 10.1126/science.1188888.

[3] M. Zimmermann, M. A. Fischbach, A family of pyrazinone natural products from a conserved nonribosomal peptide synthetase in Staphylococcus aureus, Chem. Biol. 17 (2010) 925-930. DOI :10.1016/j.chembiol.2010.08.006.
